# Integrating population variation and protein structural analysis to improve clinical interpretation of missense variation: application to the WD40 domain

**DOI:** 10.1093/hmg/ddv625

**Published:** 2016-01-05

**Authors:** Roman A. Laskowski, Nidhi Tyagi, Diana Johnson, Shelagh Joss, Esther Kinning, Catherine McWilliam, Miranda Splitt, Janet M. Thornton, Helen V. Firth, the DDD Study, Caroline F. Wright

**Affiliations:** 1 European Bioinformatics Institute (EMBL-EBI) and; 2 Wellcome Trust Sanger Institute, Wellcome Genome Campus, Hinxton, Cambridge, UK,; 3 Sheffield Regional Genetics Services, Sheffield Children's Hospital, Western Bank, Sheffield S10 2TH, UK,; 4 West of Scotland Genetic Services, Level 1, Laboratory Medicine Building, South Glasgow University Hospital, 1345 Govan Road, Glasgow G51 4TF, UK,; 5 Human Genetics, Ninewells Hospital, Dundee DD1 9SY, UK,; 6 Northern Genetics Service, Newcastle upon Tyne Hospitals NHS Foundation Trust, Institute of Genetic Medicine, International Centre for Life, Central Parkway, Newcastle upon Tyne NE1 3BZ, UK and; 7 East Anglian Medical Genetics Service, Addenbrooke's Treatment Centre, Addenbrooke's Hospital, Cambridge University Hospitals, Cambridge CB2 0QQ, UK

## Abstract

We present a generic, multidisciplinary approach for improving our understanding of novel missense variants in recently discovered disease genes exhibiting genetic heterogeneity, by combining clinical and population genetics with protein structural analysis. Using six new *de novo* missense diagnoses in *TBL1XR1* from the Deciphering Developmental Disorders study, together with population variation data, we show that the β-propeller structure of the ubiquitous WD40 domain provides a convincing way to discriminate between pathogenic and benign variation. Children with likely pathogenic mutations in this gene have severely delayed language development, often accompanied by intellectual disability, autism, dysmorphology and gastrointestinal problems. Amino acids affected by likely pathogenic missense mutations are either crucial for the stability of the fold, forming part of a highly conserved symmetrically repeating hydrogen-bonded tetrad, or located at the top face of the β-propeller, where ‘hotspot’ residues affect the binding of β-catenin to the TBLR1 protein. In contrast, those altered by population variation are significantly less likely to be spatially clustered towards the top face or to be at buried or highly conserved residues. This result is useful not only for interpreting benign and pathogenic missense variants in this gene, but also in other WD40 domains, many of which are associated with disease.

## Introduction

Understanding the impact of missense variants in known disease genes is a major challenge for the clinical application of genomics (1,2). A handful of well-known disease genes [such as *CFTR* (3) and *TP53* (4)] have been extremely well studied over several decades through both research and clinical genetic testing, and multiple known pathogenic missense variants have been individually characterized *in silico, in vitro* and *in vivo*. However, the rate of gene discovery has grown exponentially since the completion of the human genome sequence (5): nearly 3500 suspected disease genes are currently listed in OMIM, many of which have been discovered through exome sequencing of patients with rare diseases (6) with rare nonsense or protein-truncating mutations. Many such genes are, as yet, unstudied and very sparsely populated with known pathogenic (or benign) missense variants, so most rare missense variants identified in these genes are likely to be novel. Meanwhile, massively parallel sequencing technologies are increasingly being used for clinical genetic testing in the form of multigene panels, exome sequencing and even whole-genome sequencing (7). As a result, a plethora of previously uncharacterized missense variants are being discovered regularly in known disease genes (8–10), where the consequence for protein structure, cellular processes or disease aetiology is unclear, severely compromising their clinical utility.

The increasing availability of exome sequencing and whole-genome sequencing in research means that the pervasiveness of normal genetic variation is starting to become clear. A normal human genome contains three to four million variants, of which approximately 10 000 will be non-synonymous variants in coding exons predicted to cause a missense change, altering a single amino acid in the resulting protein (11,12). However, despite the fact that missense variation is extraordinarily commonplace, most genes still do not yet contain sufficient confirmed pathogenic and benign missense variants upon which to build detailed specific models to understand and accurately predict their relationship to human disease. Although numerous increasingly useful pathogenicity predictors exist (13–18), they generally have low specificity (9,19) and are based on sequence alignments that often exclude detailed knowledge of three-dimensional (3D) protein structure. However, as the same structural domain is commonly present in different proteins, encoded by different genes, and associated with different diseases, a method heavily informed by protein structure analysis is likely to yield insights across multiple genes and diseases.

Sequence data on normal population variation coupled with high throughput exome/genome sequencing of patients with rare diseases offer the perfect opportunity to investigate whether there are systematic differences between pathogenic and benign missense variants at an individual gene or protein level. Here, we use novel diagnostic *de novo* mutations identified through the Deciphering Developmental Disorders (DDD) study (20,21) as an example to explore the application of detailed protein structure analysis to the understanding of disease. As a proof of principle, we focus here on the WD40 domain, one of the most abundant structural domains in eukaryotic genomes (22). Different WD40-containing genes have already been associated with multiple diseases (23,24), including *TBL1XR1* [transducin (beta)-like 1 X-linked receptor 1], in which haploinsufficiency has recently been linked to autism spectrum disorders (25,26) and developmental delay (27–29) (OMIM no. 608628). The encoded TBL1-related protein 1 (UniProt ID Q9BZK7) is involved in a transcription signalling pathway and comprises two structural domains: an LisH domain (30) and a WD40 β-propeller domain (31). Here, we use this gene to investigate the value of integrating population variation and protein structural analysis to improve clinical interpretation of missense variation.

## Results

Six children within the DDD study were found to have likely pathogenic *de novo* mutations in *TBL1XR1*, including five single nucleotide variants predicted to cause a missense change, and one 1 bp frameshift insertion predicted to result in loss of function through truncation or nonsense-mediated decay (Table 1). Two additional likely *de novo* missense mutations have also been published in children affected by developmental disorders (25,28), as well as a *de novo* 1 bp frameshift deletion (25). A number of whole gene deletions have also been described (27,29).

**Table 1.** Summary of the clinical features in children with diagnostic variants in *TBL1XR1*

Reference	Patient ID	Age (years)	Sex	Mutation	HGVS	Clinical features	First words
DDD^a^	DECIPHER259340	11	M	*De novo* missense	ENST00000430069.1c.1322A > G ENSP00000405574.1:p.(His441Arg)	Global developmental delay	3 years
DDD^a^	DECIPHER261213	14	F	*De novo* missense	ENST00000430069.1:c.1108G > T ENSP00000405574.1:p.(Asp370Tyr)	Global developmental delay	Non-verbal
DDD^a^	DECIPHER271955	5	M	*De novo* missense	ENST00000430069.1:c.983A > G ENSP00000405574.1:p.(Asp328Gly)	Global developmental delay	Non-verbal
DDD^a^	DECIPHER273334	6	F	*De novo* missense	ENST00000430069.1:c.1331C > G ENSP00000405574.1:p.(Pro444Arg)	Global developmental delay, autism	2 years
DDD^a^	DECIPHER280701	7	M	*De novo* missense	ENST00000430069.1:c.639T > A ENSP00000405574.1:p.(His213Gln)	Global developmental delay, autism	1 year
DDD^a^	DECIPHER260965	5	M	*De novo* frameshift	ENST00000430069.1:c.800dupG ENSP00000405574.1:p.(Ile269TyrfsTer8)	Global developmental delay, autism	2–2.5 years
Saitsu *et al.* (28)	ClinVar 191371	5	F	*De novo* missense	ENST00000430069.1:c.209G > A ENSP00000405574.1p.(Gly70Asp)	Developmental delay, autistic features	Non-verbal
O'Roak *et al.* (25)	NA	Not known	F	*De novo* missense	ENST00000430069.1:c.845T > C ENSP00000405574.1:p.(Leu282Pro)	Mild/moderate IQ, autism	Unknown
O'Roak *et al.* (25)	NA	Not known	M	*De novo* frameshift	ENST00000430069.1:c.1190delT ENSP00000405574.1:p.(Ile397SerfsTer19)	Autism	Unknown
Pons *et al.* (27)	NA	8	F	Maternally inherited gene deletion	707 kb deletion (chr3:176 221 801–176 929 584)	Intellectual disability, dysmorphism (also observed in mother)	Delayed
Tabet *et al.* (29)	NA	6	F	*De novo* gene deletion	1.6 Mb deletion (chr3:175 507 453–177 095 072)	Intellectual disability, dysmorphism	2.5 years

See Supplementary Material, Table S1 for a more detailed clinical description. Variants are an notated using standard HGVS nomenclature (for simplicity, parentheses indicating missense prediction are omitted throughout the text).

^a^Variants deposited in DECIPHER database (https://decipher.sanger.ac.uk).

Children with likely pathogenic mutations in *TBL1XR1* have developmental delay often with autistic features (Table 1). All patients have marked expressive speech and language delay as the most consistent feature, and most have special needs requiring specialist educational assistance. In addition, most of the children identified via the DDD study have gastrointestinal disturbance or constipation. Although a number of patients have dysmorphic features, a preliminary assessment of facial photographs does not suggest an identifiable facial gestalt and growth parameters were typically within the normal range (Supplementary Material, Table S1). There are no apparent differences in either the phenotypes or severity of the children with missense mutations versus those with truncating mutations and gene deletions, potentially suggesting a common loss of function mechanism.

Although *TBL1XR1* is a highly constrained gene [Exome Aggregation Consortium (ExAC), Cambridge, MA, USA; http://exac.broadinstitute.org/; accessed December 2015], we were able to identify 64 unique germline population missense variants in *TBL1XR1* in population controls, in which benign variants are expected to be relatively enriched and pathogenic variants relatively depleted for rare childhood onset dominant disorders with obvious phenotypes. These variants were identified using multiple databases: the ExAC (http://exac.broadinstitute.org/; accessed June 2015), dbSNP (http://www.ncbi.nlm.nih.gov/SNP/), the Exome Variant Server [NHLBI GO Exome Sequencing Project (ESP), Seattle, WA, USA; http://evs.gs.washington.edu/EVS/; accessed June 2015] and the European Variant Archive (http://www.ebi.ac.uk/eva/) (32).

All five DDD missense mutations and one published likely pathogenic mutation are located within the WD40 domain of TBLR1, in addition to 33 of the population missense variants (Table 2). Interestingly, we also identified 16 likely non-pathogenic missense variants in *TBL1XR1* within the DDD cohort (where the variant is in, or inherited from, an unaffected parent), all of which either lie outside the WD40 domain or have already been observed in the population.

**Table 2.** All missense variants identified in *TBL1XR1* overlapping the WD40 domain of TBLR1 (June 2015; see also Fig. 4)

Variation	Source (allele count)	Location (GRCh37)	Ref/alt	Predicted amino acid change
Population	ExAC (1)	chr3:176768368	C/T	Gly153Glu
Population	dbSNP	chr3:176768338	A/G	Val163Ala
Population	ExAC (1)	chr3:176768288	C/T	Val180Ile
Population	ExAC (1)	chr3:176767892	T/A	Ser199Cys
Population	ExAC (1)	chr3:176767879	G/C	Thr203Ser
Diagnostic	DDD	chr3:176767848	A/T	His213Gln
Population	ExAC (1)	chr3:176765173	C/T	Ser260Asn
Population	dbSNP	chr3:176765158	T/C	His265Arg
Diagnostic	O'Roak *et al.* (25)	chr3:176765107	A/G	Leu282Pro
Population	ExAC (1)	chr3:176756189	T/C	Asn320Ser
Population	dbSNP	chr3:176756189	T/G	Asn320Thr
Population	EVA	chr3:176756187	T/C	Thr321Ala
Diagnostic	DDD	chr3:176756165	T/C	Asp328Gly
Population	ExAC (1)	chr3:176756102	G/T	Thr349Lys
Population	ExAC (2)	chr3:176755930	T/C	Thr360Ala
Population	dnSNP	chr3:176755930	T/A	Thr360Ser
Population	ExAC (1)	chr3:176755923	T/G	Asn362Thr
Diagnostic	DDD	chr3:176755900	C/A	Asp370Tyr
Population	ExAC (1)	chr3:176752065	T/C	Asn391Asp
Population	ExAC (2)	chr3:176752022	C/T	Gly405Glu
Population	ExAC (5)	chr3:176752016	T/C	Asn407Ser
Population	dbSNP	chr3:176752017	T/C	Asn407Asp
Population	ExAC (1)	chr3:176752014	T/C	Asn408Asp
Population	ExAC (1)	chr3:176750916	A/C	Phe420Cys
Population	ExAC (1)	chr3:176750908	T/C	Thr423Ala
Population	ExAC (1)	chr3:176750905	C/G	Val424Leu
Population	ExAC (1)	chr3:176750884	G/C	Arg431Gly
Population	dbSNP	chr3:176750883	C/T	Arg431Gln
Population	dbSNP	chr3:176750860	T/C	Thr439Ala
Population	dbSNP	chr3:176750855	T/G	Lys440Asn
Diagnostic	DDD	chr3:176750853	T/C	His441Arg
Diagnostic	DDD	chr3:176750844	G/C	Pro444Arg
Population	ExAC (1)	chr3:176750817	T/C	Asp453Gly
Population	ExAC (2)	chr3:176750811	C/T	Arg455Lys
Population	ExAC (27)	chr3:176744255	G/A	Ala475Val
Population	ExAC (1)	chr3:176744247	G/C	His478Asp
Population	ExAC (1)	chr3:176744189	T/C	Lys497Arg
Population	dbSNP	chr3:176743294	G/A	Arg513Trp
Population	ExAC (1)	chr3:176743291	T/G	Lys514Gln

**Figure 1.**
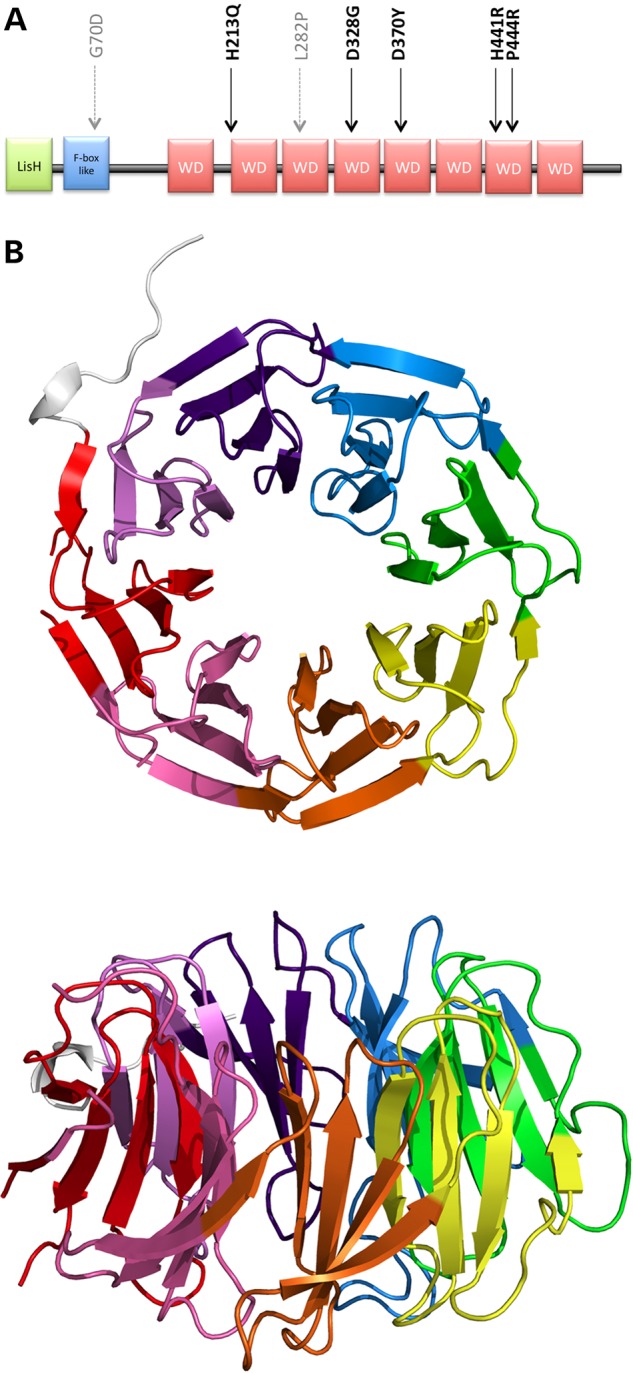
Structure of TBLR1. (**A**) Domain structure with location of diagnostic missense mutations. The five new DDD mutations are indicated in black and the two previously published mutations in grey. (**B**) Three-dimensional β-propeller structure of the WD40 domain from PDB entry 4lg9, top and side views. The eight propeller blades are rainbow coloured, starting with red for the N-terminus through to violet for the C-terminus.

**Figure 2.**
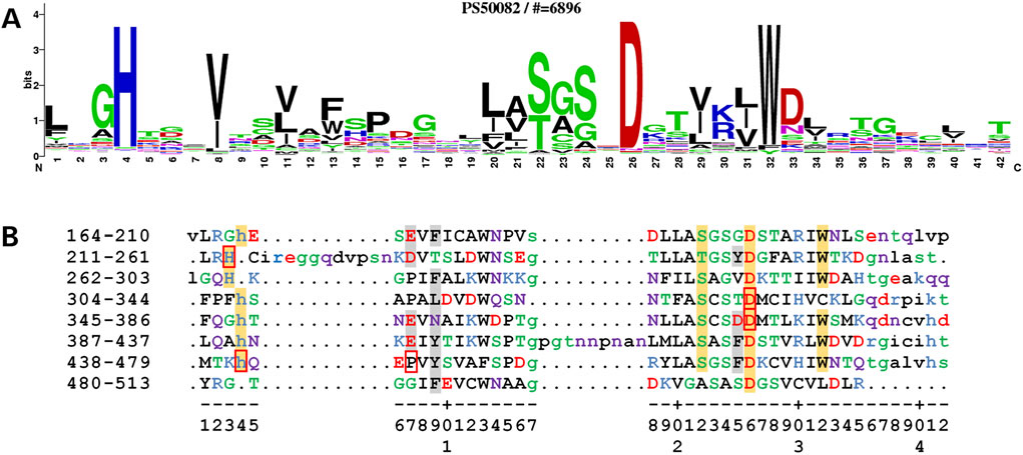
Conserved sequence elements of the WD40 motif. (**A**) PROSITE sequence logo for the WD40 motif, derived from a multiple sequence alignment of 6896 sequence fragments. The one-letter amino acid codes are coloured by type (blue basic, red acidic, green and purple polar and the rest black). The height of each corresponds to its frequency of occurrence in the alignment. (**B**) Structure-based alignment of the eight WD40 motifs in the crystal structure of TBLR1. The motifs were manually extracted from the 4lg9 PDB file and then aligned using the PDBeFold Server (36). The numbers on the left show the range of residue numbers in the sequence on that line. The one-letter amino acid codes are coloured as per the PROSITE sequence logo (A); lower-case letters correspond to residues not aligned in the 3D superposition. The numbers along the bottom roughly correspond to the sequence positions in the WD40 motif in (A). The amino acids having an orange background are those belonging to the Asp-His-Ser/Thr-Trp tetrad. The red borders identify the five amino acids involved in the DDD missense mutations: His213Gln, Asp328Gly, Asp370Tyr, His441Arg and Pro444Arg. The amino acids with the light grey backgrounds are the hotspot residues on the domain's top face, as identified by WDSPdb (31), being the ones likely to interact with β-catenin when it binds.

**Figure 3.**
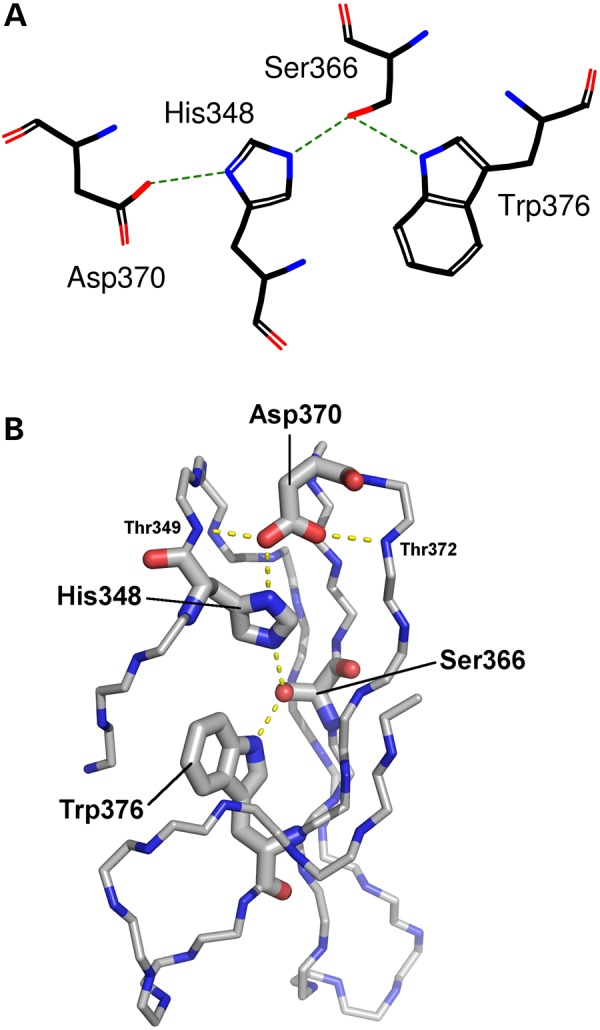
Representation of the hydrogen-bonding network of the DHSW tetrad. Taken from the fifth WD40 motif in the 3D structure of TBLR1 (PDB entry 4lg9). (**A**) Schematic representation showing the four sidechains involved: Asp370, His348, Ser366 and Trp376. Hydrogen bonds are shown by the green dotted lines. (**B**) Three-dimensional representation showing the location and sidechains of the four tetrad residues; the rest of the domain is represented only by backbone atoms N, C^α^ and C. Potential hydrogen bonds are shown by the dashed lines. Note the importance of the highly conserved Asp370, which can not only hydrogen-bond to the histidine, but also to the backbone of neighbouring strands, helping hold the propeller-blade structure together.

The WD40 domain of TBLR1 has a β-propeller structure consisting of eight propeller ‘blades’, each formed by a four-stranded antiparallel β-sheet, which are joined by β-hairpins. The blades are arranged symmetrically about a central axis, like the staves of a barrel, and β-catenin binds to the ‘top’ face of the propeller to promote the transcription of Wnt target genes (33) (Fig. 1B). A number of ‘hotspot residues’ have been identified previously (31) on the top face of the domain (34), which are likely to be involved in the protein's interaction with β-catenin. In addition, the amino acid sequence of each blade of the β-propeller in most WD40 domains, including that in TBLR1, exhibits a recognizable pattern of residues known as the WD40 repeat motif, with certain residue types favoured in specific positions. The PROSITE sequence logo (35) for this motif is shown in Figure 2A, in which taller letters identify the highly conserved residues that are important for stabilization of the blade's structure. The TBLR1 protein has six complete tetrads and one incomplete tetrad that is missing the tryptophan residue (Fig. 2B). Of note in the logo are the histidine, serine/threonine, aspartic acid and tryptophan residues at motif positions 4, 22, 26 and 32, respectively. These form the Asp-His-Ser/Thr-Trp (DHSW) tetrad—a network of ‘unusually strong’ hydrogen bonds that maintains the domain's thermostability (37) (Fig. 3). The aspartic acid at motif position 26 is present in all eight blades and plays an especially important role in stabilizing the beta-hairpin structure at the top of each blade via two hydrogen bonds to the main chain nitrogen atoms of adjoining strands. An experimental study in 2010 showed that, although mutations to the tetrad residues maintained the domain's 3D structure, as evidenced by crystal structures of the mutant proteins, the stability of the proteins was severely affected (37), potentially interfering with folding or function.

**Figure 4.**
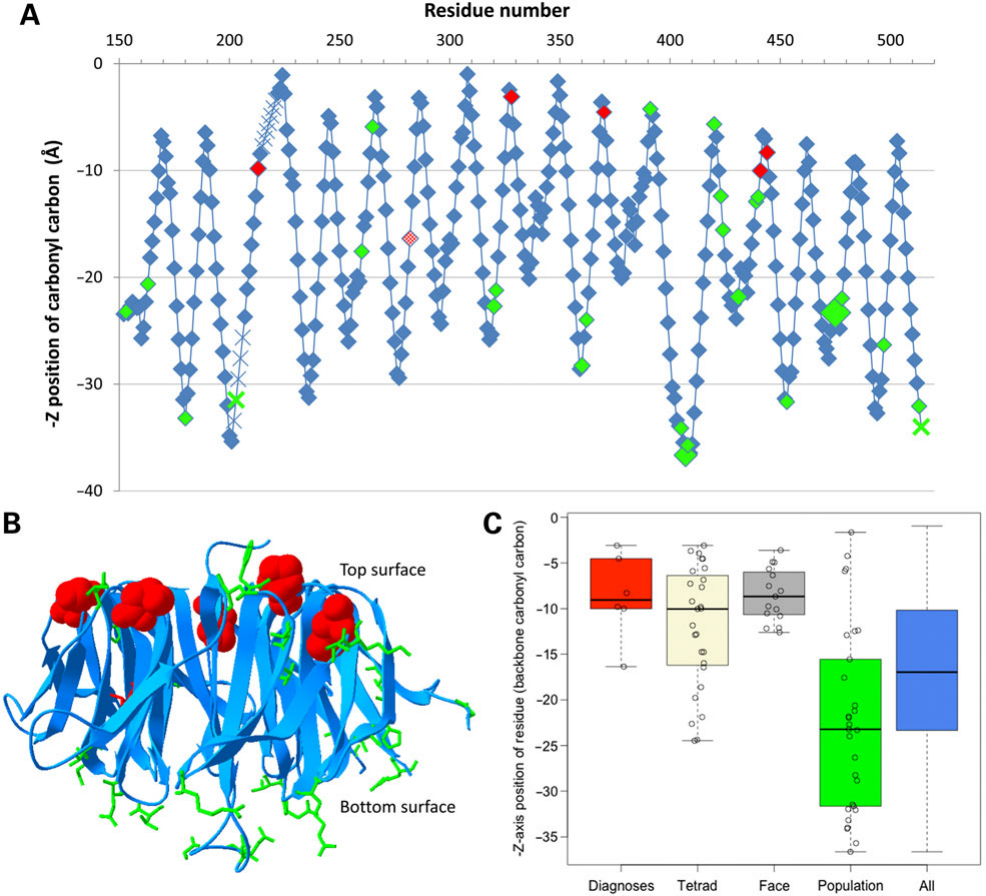
*Z*-axis location of all variants in the WD40 domain of TBLR1. (**A**) Graphical representation taken from the top to bottom face (PDB entry 4lg9). Likely pathogenic missense mutations are indicated in red (with new diagnoses from the DDD study completely filled), whereas population missense variants are indicated in green and other residues are indicated in blue. The backbone position of all residues is shown, based on the *Z*-axis location of the backbone carbonyl carbon in the crystal structure. Larger diamonds represent variants that are present multiple times across the databases, and crosses indicate the approximate interpolated location of residues that are absent from the PDB file. (**B**) Three-dimensional representation viewed from the side using PDB entry 4lg9, with all missense variants highlighted using-stick representation (space-filled for new DDD diagnoses). Likely pathogenic missense mutations are indicated in red, whereas population missense variants are indicated in green and the rest of the domain is represented using blue ribbons. (**C**) Boxplot of *Z*-axis location in PDF entry 4lg9 of diagnostic mutations (red), the conserved tetrads (beige), hotspot residues on the top face (grey), population variation (green) and all amino acid residues in the domain (blue) in the TBLR1 protein. *P*-values are not significant between the diagnostic/tetrad/top face residues or between population/all residues, but are significant between these groups (diagnostic versus population residues, *P* = 9×10^−5^).

The five DDD missense mutations are His213Gln, Asp328Gly, Asp370Tyr, His441Arg and Pro444Arg. The first four involve histidine and aspartic acid residues from different symmetrically repeated DHSW tetrads, at positions 4 and 26 in the WD40 motif (Fig. 2B), so that their change is likely to disrupt the stability of the protein's fold. Of particular interest is the highly conserved aspartic acid at position 26 in the WD40 motif, which can hydrogen-bond to the tetrad's histidine and also to a main-chain nitrogen on the preceding propeller blade (Thr349 in Fig. 3), and to a main-chain nitrogen two residues down (Thr372 in Fig. 3). In their native state, both are structurally stabilizing interactions, helping to hold the propeller together. The latter interaction helps maintain the beta turn that joins the two beta strands either side of the Asp. The only non-DDD likely pathogenic missense mutation identified is Leu282Pro, which is at position 21 in the WD40 motif, adjacent to a DHSW tetrad, where addition of a proline residue likely alters the packing of the strands sufficiently to alter the hydrogen bond network inside the tetrad. The fifth of the DDD mutations, Pro444Arg, occurs at position 7 in the WD40 motif (Fig. 2B). This is not a highly conserved position, although there are three proline residues at this position in TBLR1. Here, the fact that the amino acid is on the domain's top face (Fig. 4), coupled with the dramatic nature of the change, is likely to be responsible for the deleterious effect of the mutation. The mutation places a large, charged arginine at the protein–protein interface, and this potentially interferes with, or disrupts, the interaction required for the protein's function.

To evaluate the structural impact of the missense mutations in this domain further, the six amino acids with likely pathogenic missense mutations in the WD40 domain were compared with the 29 amino acids with benign population missense variation (Table 2). The location of these amino acids along the *Z*-axis of the protein structure in PDB entry 4lg9 was analysed, i.e. through the middle of the β-barrel, from the top to bottom face (Fig. 4A and B), indicating that the disease-associated amino acids are clustered in 3D space and significantly different—closer to the top binding face—from those associated with presumed benign variation (*P* = 9 × 10^−5^, Fig. 4C). In addition, disease-associated amino acids were also predicted to be significantly different from those associated with benign variation using PolyPhen (15) (*P* = 2 × 10^−8^), SIFT (18) (*P* = 7 × 10^−4^), solvent-exposed surface area (38) (*P* = 2 × 10^−7^) and residue conservation (39) (*P* = 8 × 10^−4^), but did not differ significantly from the tetrad or top face hotspot residues.

## Discussion

We have used the 3D structure of the WD40 domain encoded by the gene *TBL1XR1* to understand and characterize the differences between likely pathogenic *de novo* missense mutations detected in children with severe developmental delay and presumed benign missense variation seen in population samples and the ExAC data set. Although the variants are predicted to result in missense changes, the true biological effect on the resulting protein is unknown. As has been observed previously across all proteins (40), the likely pathogenic mutations in TBLR1 are generally at more buried and conserved sites when compared with population variation. When the structure of the WD40 domain of this protein is considered in detail, there is notable clustering in 3D space, with likely pathogenic mutations more likely to be near the top face of the domain. Specifically, likely pathogenic mutations in TBLR1 all affect either the structural rigidity of the WD40 domain β-propeller, compromising the stability of the fold, or the physicochemical characteristics of the top face of the β-propeller, affecting the binding of β-catenin. The overlap of these diagnostic variants with the previously identified symmetrically repeating DHSW tetrads and top face hotspot residues (34) allows us to make strong predictions about the location of other likely pathogenic genetic variations both in TBLR1 and in other instances of this domain.

The WD40 domain is one of the top 10 most abundant domains in eukaryotic genomes, although rarely present in prokaryotes (22). Its primary role appears to be in making protein–protein interactions, which it can make simultaneously with several different proteins, particularly in relation to forming and regulating protein, DNA or RNA complexes (22,41). A number of diseases are known to be associated with mutations in WD40 domains (23,24), including numerous developmental phenotypes such as lissencephaly (42), short-rib thoracic dysplasia (43) and reduced neuronal migration (44). Twenty-one proteins containing such disease-associated mutations are listed in Supplementary Material, TableS2, with their corresponding locations in the WD40 motif.

As next generation sequencing of gene panels and whole exomes/genomes is increasingly applied in both research and clinical settings, more and more benign and likely pathogenic missense variants will be uncovered in known disease genes as well as in novel disease genes. Although *in silico* predictions alone should not be relied on as the sole basis to determine the clinical significance of missense variants in proteins, we hope that the analysis used in this study provides useful structural evidence for variant interpretation. Moreover, combining clinical and population genetics with protein structural analysis offers widely applicable *in silico* method for improving the clinical interpretation of novel missense variation.

## Materials and Methods

The DDD study was approved by the UK Research Ethics Committee (10/H0305/83, granted by the Cambridge South REC, and GEN/284/12 granted by the Republic of Ireland REC), and appropriate informed consent was obtained from all participants. Patients meeting the recruitment criteria (neurodevelopmental disorder and/or congenital anomalies, abnormal growth parameters, dysmorphic features and unusual behavioural phenotypes) were recruited to the DDD study (www.ddduk.org) by their UK NHS and Republic of Ireland Regional Genetics Service, who also recorded clinical information and phenotypes using the Human Phenotype Ontology (45) via a secure web portal within the DECIPHER database (46). DNA samples from patients and their parents were analysed by the Wellcome Trust Sanger Institute using high-resolution microarray analysis (array-CGH and SNP-genotyping) to investigate copy number variations in the child and by exome sequencing to investigate single nucleotide polymorphisms and small insertions/deletions (indels). Putative *de novo* sequence variants of interest were validated in-house using either targeted Sanger sequencing or MiSeq sequencing. All genomic variants were annotated with the most severe consequence predicted by Ensembl Variant Effect Predictor (47) and their minor allele frequencies observed in diverse population samples. As has been described previously (20), likely diagnostic variants were fed back to referring clinical geneticists for validation in an accredited diagnostic laboratory and discussion with the family via patients’ record in DECIPHER, where they can be viewed in an interactive genome browser.

In a data set of the first 4295 family trios (child, mother and father) with exome sequence data, we investigated genes already robustly implicated in developmental disorders with more than three *de novo* mutations in DDD children, where the consequence was predicted to result in different missense changes. We cross-referenced this list against the Protein Data Bank (48) to limit our analysis to genes with solved protein structures and further refined the list to those where all missense changes lay within a high-quality crystal structure from the human-derived protein. We further excluded metalloproteins and enzymes in which the missense variants clustered in the catalytic site, and here we limit our discussion to just one gene, *TBL1XR1*, a fairly recently identified developmental disorder gene (25–29), in which multiple likely pathogenic missense mutations were found in DDD that map onto a 3D protein domain structure.

Additional causal variants in *TBL1XR1* in children with autism/developmental delay were identified through ClinVar (http://www.ncbi.nlm.nih.gov/clinvar/) (49) and a search of published literature. Population variation in this gene was also investigated using the ExAC (http://exac.broadinstitute.org/; accessed June 2015), dbSNP (http://www.ncbi.nlm.nih.gov/SNP/), the Exome Variant Server (NHLBI GO ESP; http://evs.gs.washington.edu/EVS/; accessed June 2015) and the European Variant Archive (http://www.ebi.ac.uk/eva/) (32).

## Supplementary Material

Supplementary Material is available at *HMG* online.

## Funding

This work was supported by the Health Innovation Challenge Fund (grant no. HICF-1009-003), a parallel funding partnership between the Wellcome Trust and the Department of Health and the Wellcome Trust Sanger Institute (grant no. WT098051). The views expressed in this publication are those of the author(s) and not necessarily those of the Wellcome Trust or the Department of Health. The study has UK Research Ethics Committee approval (10/H0305/83, granted by the Cambridge South REC, and GEN/284/12, granted by the Republic of Ireland REC). Funding to pay the Open Access publication charges for this article was provided by the Wellcome Trust Sanger Institute.

## Supplementary Material

Supplementary Data
