# Supplementary material for: Integrating population variation and protein structural analysis to improve clinical interpretation of missense variation: application to the WD40 domain
Source: Hum Mol Genet. 2016 Jan 5;25(5):927–35. doi: 10.1093/hmg/ddv625 (PMC4754046; doi:10.1093/hmg/ddv625)
Supplement: Supplementary Data [file supp_25_5_927__index.html]

Integrating population variation and protein structural analysis to improve clinical interpretation of missense variation: application to the WD40 domain — Integrating population variation and protein structural analysis to improve clinical interpretation of missense variation: application to the WD40 domain — Supplementary Data 

# Integrating population variation and protein structural analysis to improve clinical interpretation of missense variation: application to the WD40 domain

## Supplementary Data

Supplementary Data

- Supplementary Table 1 - xlsx file
- Supplementary Table 2 - docx file
