# Supplementary material for: Integrating population variation and protein structural analysis to improve clinical interpretation of missense variation: application to the WD40 domain
Source: Hum Mol Genet. 2016 Jan 5;25(5):927–35. doi: 10.1093/hmg/ddv625 (PMC4754046; doi:10.1093/hmg/ddv625)
Supplement: Supplementary Data [file supp_ddv625_ddv625supp_table2.docx]

| **Gene** | **UniProt** | **Protein** | **Mutation** | **Pos^a^** | **Disease** | **OMIM** | **Ref** |
| --- | --- | --- | --- | --- | --- | --- | --- |
| **AAAS** | Q9NRG9 | Aladin | His160Arg  Ser263Pro | 14  24 | Achalasia-addisonianism-alacrima syndrome  “ “ “ “ | 231550  “ | [^1^](#_ENREF_14) |
| **AHI1** | Q8N157 | Jouberin | Arg723Gln | 30 | Joubert syndrome 3 | 608629 | [^2^](#_ENREF_20) |
| **COPA** | P53621 | Coatomer subunit alpha | Lys230Asn  Arg233His | 30  34 | Autoimmune interstitial lung, joint, and kidney disease  “ “ “ “ “ | 616414 | ^3^ |
| **CORO1A** | P31146 | Coronin-1A | Val134Met | 8 | Immunodeficiency | 615401 | [^4^](#_ENREF_1) |
| **DDB2** | Q92466 | DNA damage-binding protein 2 | Lys244Glu  Arg273His | 7  35 | Xeroderma pigmentosum complementation group E  “ “ “ “ “ | 278740 | [^5^](#_ENREF_22) |
| **DNAI1** | Q9UI46 | Dynein intermediate chain 1, axonemal | Gly515Ser | 39 | Kartagener syndrome | 244400 | ^6^ |
| **ERCC8** | Q13216 | DNA excision repair protein ERCC-8 | Trp194Cys  Leu202Ser  Ala205Pro  Trp361Cys | 13  20  23  32 | Cockayne syndrome A  “ “  “ “  UV-sensitive syndrome 2 | 216400  “  “ 614621 | [^7^](#_ENREF_9)  “  [^8^](#_ENREF_10)  [^9^](#_ENREF_11) |
| **GNB4** | Q9HAV0 | Guanine nucleotide-binding protein subunit beta-4 | Gly53Asp  Lys89Glu | 3  39 | Charcot-Marie-Tooth disease, dominant, intermediate type, F | 615185 | [^10^](#_ENREF_18) |
| **IFT140** | Q96RY7 | Intraflagellar transport protein 140 homolog | Val292Met | 21 | Short-rib thoracic dysplasia 9 with or without polydactyly | 266920 | ^11^ |
| **IFT80** | Q9P2H3 | Intraflagellar transport protein 80 homolog | His105Gln | 4 | Short-rib thoracic dysplasia 2 | 611263 | [^12^](#_ENREF_12) |
| **KATNB1** | Q9BVA0 | Katanin p80 WD40 repeat-containing subunit B1 | Gly33Trp | 18 | Lissencephaly 6, with microcephaly | 616212 | ^13^ |
| **LRBA** | P50851 | Lipopolysaccharide-responsive and beige-like anchor protein | Ile2657Ser | 20 | Immunodeficiency, common variable, 8, with autoimmunity (CVID8) | 614700 | [^14^](#_ENREF_8) |
| **PAFAH1B1** | P43034 | Lissencephaly-1 protein | His149Arg  Gly162Ser  Ser169Pro  Arg241Pro  His277Pro  Asp317His | 4  17  24  12  6  26 | Lissencephaly  “  “  Subcortical band heterotopias  Lissencephaly  Reduces neuronal migration | 607432  “  “  “  “  “ | [^15^](#_ENREF_2)  [^16^](#_ENREF_3)  [^17^](#_ENREF_4)  [^18^](#_ENREF_5)  [^19^](#_ENREF_6)  [^20^](#_ENREF_7) |
| **PEX7** | O00628 | Peroxisomal targeting signal 2 receptor | Gly217Arg  Ala218V | 23  24 | Rhizomelic chondrodysplasia punctata 1  “ | 614879 | [^21^](#_ENREF_19) |
| **POC1A** | Q8NBT0 | POC1 centriolar protein homolog A | Leu171Pro | 31 | Short stature, onychodysplasia, facial dysmorphism, and hypotrichosis | 614813 | [^22^](#_ENREF_16) |
| **POC1B** | Q8TC44 | POC1 centriolar protein homolog B | Arg106Pro | 9 | Cone-rod dystrophy 20 | 615973 | [^23^](#_ENREF_15) |
| **PRPF4** | O43172 | U4/U6 small nuclear ribonucleoprotein Prp4 | Pro315Leu | 39 | Retinitis pigmentosa 70 | 615922 | [^24^](#_ENREF_17) |
| **THOC6** | Q86W42 | THO complex subunit 6 homolog | Gly46Arg | 23 | Beaulieu-Boycott-Innes syndrome | 613680 | ^25^ |
| **WDR34** | Q96EX3 | WD repeat-containing protein 34 | Ala341Val  Thr354Met  Pro390Leu  Gly393Ser  Ser410Ile  Lys436Arg  Arg447Gln  Arg447Trp | 28  41  3  6  22  6  18  18 | Short-rib thoracic dysplasia 11  “ “ “  “ “ “  “ “ “  “ “ “  “ “ “  “ “ “  “ | 615633  “  “  “  “  “  “  “ | [^26^](#_ENREF_13) |
| **WDR36** | Q8NI36 | WD repeat-containing protein 36 | Asn355Ser  Ala449Thr  Arg529Gln  Asp658Gly | 27  4  40  40 | Glaucoma 1, open angle, G  “ “ “  “ “ “  “ “ “ | 609887  “  “  “ | [^27^](#_ENREF_21) |
| **WRAP53** | Q9BUR4 | Telomerase Cajal body protein 1 | His376Tyr  Arg398Trp  Gly435Arg | 4  35  27 | Dyskeratosis congenita, autosomal recessive, 3  “ “ “ “ “  “ “ “ “ “ | 613988  “  “ | ^28^ |

**Supplementary Table 2.** Twenty-one proteins containing disease-associated mutations in the WD40 motif, with the corresponding locations of the mutations in the motif.

*Position in the WD40 domain (Figure 2)

**References**

1. Handschug, K. et al. Triple A syndrome is caused by mutations in AAAS, a new WD-repeat protein gene. *Human Molecular Genetics* **10**, 283-90 (2001).

2. Valente, E.M. et al. AHI1 gene mutations cause specific forms of Joubert syndrome-related disorders. *Annals of Neurology* **59**, 527-34 (2006).

3. Watkin, L.B. et al. COPA mutations impair ER-Golgi transport and cause hereditary autoimmune-mediated lung disease and arthritis. *Nature Genetics* **47**, 654-60 (2015).

4. Moshous, D. et al. Whole-exome sequencing identifies Coronin-1A deficiency in 3 siblings with immunodeficiency and EBV-associated B-cell lymphoproliferation. *Journal of Allergy and Clinical Immunology* **131**, 1594-603 (2013).

5. Hwang, B.J., Toering, S., Francke, U. & Chu, G. p48 Activates a UV-damaged-DNA binding factor and is defective in xeroderma pigmentosum group E cells that lack binding activity. *Molecular and Cellular Biology* **18**, 4391-9 (1998).

6. Guichard, C. et al. Axonemal dynein intermediate-chain gene (DNAI1) mutations result in situs inversus and primary ciliary dyskinesia (Kartagener syndrome). *American Journal of Human Genetics* **68**, 1030-5 (2001).

7. Laugel, V. et al. Mutation update for the CSB/ERCC6 and CSA/ERCC8 genes involved in Cockayne syndrome. *Human Mutation* **31**, 113-26 (2010).

8. Cao, H., Williams, C., Carter, M. & Hegele, R.A. CKN1 (MIM 216400): mutations in Cockayne syndrome type A and a new common polymorphism. *Journal of Human Genetics* **49**, 61-3 (2004).

9. Nardo, T. et al. A UV-sensitive syndrome patient with a specific CSA mutation reveals separable roles for CSA in response to UV and oxidative DNA damage. *Proceedings of the National Academy of Sciences of the United States of America* **106**, 6209-14 (2009).

10. Soong, B.W. et al. Exome sequencing identifies GNB4 mutations as a cause of dominant intermediate Charcot-Marie-Tooth disease. *American Journal of Human Genetics* **92**, 422-30 (2013).

11. Perrault, I. et al. Mainzer-Saldino syndrome is a ciliopathy caused by IFT140 mutations. *American Journal of Human Genetics* **90**, 864-70 (2012).

12. Beales, P.L. et al. IFT80, which encodes a conserved intraflagellar transport protein, is mutated in Jeune asphyxiating thoracic dystrophy. *Nature Genetics* **39**, 727-9 (2007).

13. Hu, W.F. et al. Katanin p80 regulates human cortical development by limiting centriole and cilia number. *Neuron* **84**, 1240-57 (2014).

14. Lopez-Herrera, G. et al. Deleterious mutations in LRBA are associated with a syndrome of immune deficiency and autoimmunity. *American Journal of Human Genetics* **90**, 986-1001 (2012).

15. Lo Nigro, C. et al. Point mutations and an intragenic deletion in LIS1, the lissencephaly causative gene in isolated lissencephaly sequence and Miller-Dieker syndrome. *Human Molecular Genetics* **6**, 157-64 (1997).

16. Leventer, R.J., Cardoso, C., Ledbetter, D.H. & Dobyns, W.B. LIS1 missense mutations cause milder lissencephaly phenotypes including a child with normal IQ. *Neurology* **57**, 416-22 (2001).

17. Feng, Y. et al. LIS1 regulates CNS lamination by interacting with mNudE, a central component of the centrosome. *Neuron* **28**, 665-79 (2000).

18. Sicca, F. et al. Mosaic mutations of the LIS1 gene cause subcortical band heterotopia. *Neurology* **61**, 1042-6 (2003).

19. Torres, F.R. et al. Mutation screening in a cohort of patients with lissencephaly and subcortical band heterotopia. *Neurology* **62**, 799-802 (2004).

20. Tanaka, T. et al. Lis1 and doublecortin function with dynein to mediate coupling of the nucleus to the centrosome in neuronal migration. *Journal of Cell Biology* **165**, 709-21 (2004).

21. Braverman, N. et al. Human PEX7 encodes the peroxisomal PTS2 receptor and is responsible for rhizomelic chondrodysplasia punctata. *Nature Genetics* **15**, 369-76 (1997).

22. Sarig, O. et al. Short stature, onychodysplasia, facial dysmorphism, and hypotrichosis syndrome is caused by a POC1A mutation. *American Journal of Human Genetics* **91**, 337-42 (2012).

23. Roosing, S. et al. Disruption of the basal body protein POC1B results in autosomal-recessive cone-rod dystrophy. *American Journal of Human Genetics* **95**, 131-42 (2014).

24. Chen, X. et al. PRPF4 mutations cause autosomal dominant retinitis pigmentosa. *Human Molecular Genetics* **23**, 2926-39 (2014).

25. Beaulieu, C.L. et al. Intellectual disability associated with a homozygous missense mutation in THOC6. *Orphanet Journal of Rare Diseases* **8**, 62 (2013).

26. Schmidts, M. et al. Mutations in the gene encoding IFT dynein complex component WDR34 cause Jeune asphyxiating thoracic dystrophy. *American Journal of Human Genetics* **93**, 932-44 (2013).

27. Monemi, S. et al. Identification of a novel adult-onset primary open-angle glaucoma (POAG) gene on 5q22.1. *Human Molecular Genetics* **14**, 725-33 (2005).

28. Zhong, F. et al. Disruption of telomerase trafficking by TCAB1 mutation causes dyskeratosis congenita. *Genes and Development* **25**, 11-6 (2011).
